# Investigating the Effects of *Arabidopsis thaliana* Cruciferin Double Knockouts on Amino Acid Profiles, Dry Seed Proteome, and Oxidative Stress Levels

**DOI:** 10.17912/micropub.biology.001441

**Published:** 2025-04-14

**Authors:** Clement Bagaza, Huda Ansaf, Abou Yobi, Ruthie Angelovici

**Affiliations:** 1 Division of Biological Sciences, University of Missouri, Columbia, Missouri, United States

## Abstract

As plant seeds mature, they accumulate large quantities of seed storage proteins, which are a vital source of carbon, nitrogen, and sulfur necessary for establishing the seedling, especially during the transition from the heterotrophic to the photoautotrophic stage. However, seed storage proteins in many crop seeds are deficient in essential amino acids, which cannot be synthesized by humans and monogastric animals and must be obtained from the diet. Lysine and tryptophan are the most deficient amino acids in cereal seeds, while methionine is the most deficient amino acid in legumes. In the last few decades, extensive research has been done to improve the nutritional quality of seed crops. However, much of this effort was hindered due to the conserved natural phenomenon of proteomic rebalancing that ‘resets’ the seed’s protein-bound amino acid composition despite major alterations to the proteomic sink. Neither the underlying regulatory mechanism nor the natural function of proteomic rebalancing is well understood. To address this gap, we used the model organism *Arabidopsis thaliana* to investigate the impact of cruciferin (CRU) seed storage protein double knockouts on key biological processes. Amino acid analysis showed that the protein-bound amino acid composition and levels did not change in the mutants despite major alterations in the proteome, especially in the double mutant lacking both CRUA and CRUC ( *cruac* ). This mutant also has the highest free amino acid changes and experienced the most oxidative stress damage compared to other mutants based on analysis of protein carbonylation and glutathione levels. The mutant that lacks CRUA and CRUB ( *cruab* ), on the other hand, was the least affected in all the traits examined. These results suggest that CRUs are not functionally redundant, and that each CRU is not replaceable by another in *Arabidopsis* . The results also show that *Arabidopsis* seed protein-bound amino acid composition is fully rebalanced in the double CRU mutants despite major proteome alteration.

**Figure 1. Metabolomics and proteomics characterization of double knockout mutants of the seed storage proteins, cruciferins:**
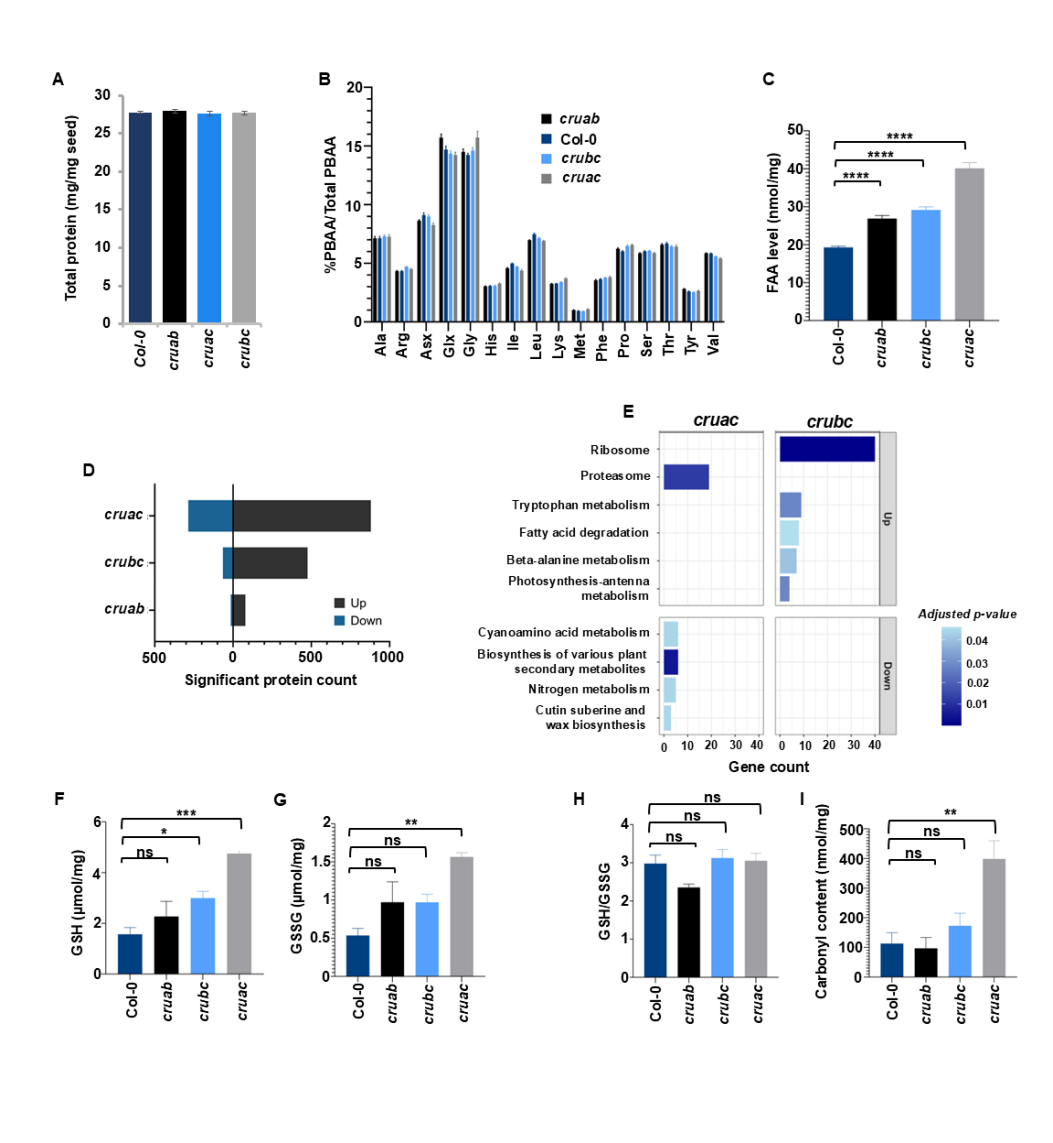
**A** . Total protein content in the dry seed measured from Col-0 and the double mutants (n = 3). **B.** Total protein-bound amino acids (TPBAA) composition (%PBAA/TPBAA) in the dry seed measured from Col-0 and the double mutants (n = 6). **C** , Total free amino acids (TFAA) levels (nmol/mg) measured from Col-0 and the double mutants at dry seed stage (n=6). **D** , Bar graph that shows the number of DEPs that increased (gray) or decreased (blue) in each mutant compared to Col-0. **E** , KEGG pathway analysis for DEPs of *cruac* and *crubc* mutants. The enrichment analysis of DEPs for the *cruab* mutant did not yield any significant results. **F** , Reduced glutathione (GSH) levels in the dry seed (n = 4). G, Oxidized glutathione (GSSG) levels in the dry seed (n = 4). **H** , The ratio between GSH and GSSG (n = 4). **I** , Carbonyl content in the dry seed (n = 3). Error bars represent standard error of the mean. Statistical analyses were performed using one-way ANOVA with Dunnett's multiple comparisons test. Statistically significant values are indicated by star (*): * p < 0.05; ** p < 0.01; p < 0.001; ns, not significant.

## Description

Seeds of staple crops are an important nutritional source but are insufficient to meet our dietary requirements (Larkins et al., 2017). Despite our reliance on them in our diets, they are deficient in several essential amino acids (EAA), which are amino acids that humans and other vertebrates, including farm animals, cannot synthesize in their bodies and, therefore, must be obtained from their diet (Hou and Wu, 2018). While individual plant seeds may be deficient in certain EAAs, legumes and cereals complement each other to form a more balanced protein source. For example, lysine (Lys) and tryptophan (Trp) are the most deficient amino acids in cereal seeds, while methionine (Met) is the most deficient amino acid in legumes (Boyer *et al.* , 1992, Shewry and Halford, 2002, Galili *et al.* , 2005, Shewry, 2007, Ufaz and Galili, 2008). Therefore, humans and/or farm animals that rely on a plant-based diets, mainly cereals, must be supplemented with additional costly protein sources. One of the main culprits causing low protein quality in legumes and cereals is the high abundance of seed storage proteins (SSPs; 50-70%), which are poor in EAA. The three main types of SSPs are prolamins, globulins, and albumins (Shewry and Halford, 2002). Since 91-99% of the total amino acids in seeds reside in proteins (Amir et al., 2018), much effort was centered on identifying SSP mutants. Different SSP mutants have been generated for different crop species (Mertz et al., 1964; Kawakatsu and Takaiwa, 2010; Li et al., 2018). Surprisingly, despite the massive perturbation and reprogramming of the seed proteome in the various SSPs mutants, the seeds' overall relative protein-bound amino acid (PBAA) composition remained highly comparable to the unperturbed cultivars (Wu and Messing, 2014). Since the overall seed PBAA composition and protein content remained largely unchanged compared to the unperturbed lines, this suggests a tightly regulated phenotype. This natural phenomenon was termed proteomic rebalancing and is highly conserved in seeds having been reported not only in maize (Morton et al., 2016) but also in soybean ( *Glycine max* ; (Schmidt et al., 2011), *Arabidopsis thaliana* (Withana-Gamage et al., 2013), camelina (Schmidt and Pendarvis, 2017), and wheat (Altenbach et al., 2014). Neither the underlying regulatory mechanism nor the natural function of proteomic rebalancing is well understood. We mainly know that it is characterized by a substantial re-programming of non-SSPs (Bagaza et al., 2024), compensating for the protein content levels and the seed’s overall amino acid composition.

Since proteomic rebalancing is a conserved mechanism observed across various monocot and dicot seed types, *Arabidopsis thaliana* is an ideal model system for investigating the molecular machinery underlying this process. Its genetic tractability and extensive functional resources make it particularly suited for uncovering the regulatory pathways that control proteomic rebalancing. The SSPs in *Arabidopsis thaliana* predominantly comprise the 12S globulin (CRU) and 2S albumin (napin) types, with lesser contributions from oleosins, defensins, and late embryogenesis abundant proteins (Wanasundara, 2011). CRU is the predominant protein and is an 12S hexameric globulin with a molecular weight of 300–360 kDa and belongs to the cupin superfamily (Wan et al., 2007; Perera et al., 2016). *Arabidopsis* has four paralogous genes that encode CRU proteins, At5g44120.3 (CRUA), At1g03880.1 (CRUB), At4g28520.1 (CRUC), and At1g3890.1 (CRUD) (Wan et al., 2007). Each cruciferin subunit consists of a heavy acidic (α) chain and a light basic (β) chain, connected by a disulfide bond (Withana-Gamage et al., 2013).

The CRUs of *A. thaliana* are closely related to the economically significant oilseed species, *Brassica napus* (Cavell et al., 1998). In *B. napus* , CRUs constitute approximately 60% of the total dry seed proteome (Perera et al., 2016). In *Arabidopsis* , CRUs are dominated by three proteins (Pang et al., 1988; Li et al., 2007; Wan et al., 2007): CRUA, CRUB, and CRUC, although a fourth less abundant paralog, CRUD, is also a member of this family (Pang et al., 1988; Li et al., 2007; Wan et al., 2007). In our most recent publication, we have established a link between PBAA rebalancing and the alterations in translation and redox state in seeds devoid of the three CRU proteins (triple-knockout; *cruabc* ) (Bagaza et al., 2024). We also examined single CRU mutants and their effects on dry seed proteome. Our results showed little redundancy among the mutants (Bagaza et al., 2024).

In this study, we utilized three previously generated and validated *Arabidopsis thaliana* lines, each expressing only one CRU while the other two are knocked out ( *cruab* , *cruac* , and *crubc* , which express CRUC, CRUB, and CRUA, respectively). These double mutants were generated by crossing the single T-DNA mutants. The single T-DNA mutants, generated and validated by (Withana-Gamage et al., 2013), correspond to Salk_00266 ( *crua* ), Salk_045987) ( *crub* ), and GABI_283D09 *(cruc* ). While the triple knockout completely removes all three main CRUs, enabling a broad assessment of their elimination, studying double mutants provides a more detailed and mechanistic understanding of how specific mutant combinations contribute to proteomic rebalancing and other essential seed functions.

First, we measured the total protein content using the Pierce 660 kit (Thermo Scientific). No significant differences in total amino acid content were observed between any mutant and the wild type, Col-0 ( **Figure 1A** ). We then compared the PBAA and free amino acid (FAA) levels and composition changes between the mutants and Col-0. PBAAs were measured using a combination of acid hydrolysis and an ultra-performance liquid chromatography-tandem mass spectrometer (UPLC-MS/MS) instrument (Waters Corporation, Milford, MA) approach as detailed in (Yobi et al., 2020; Bagaza et al., 2024). With acid hydrolysis, Trp and cysteine (Cys) are lost. In addition, asparagine (Asn) and glutamine (Gln) are recovered as aspartate (Asp) and glutamate (Glu), respectively. Hence, the combination of Asn and Asp is denoted Asx, while the combination of Gln and Glu is denoted Glx. The remaining steps of the PBAA analysis were the same as for the FAA analysis, which was based on water extraction as described in (Yobi et al., 2020; Bagaza et al., 2024). In total, we quantified 16 PBAAs that represent 18 amino acids and all 20 proteogenic FAAs ( **Amino Acid Dataset A** ). We calculated the amino acid relative composition, which is the percentage of each amino acid level (nmol/mg) to the sum of all the amino acids measured ( **Amino Acid Dataset B)** . No amino acid relative composition changed (i.e., individual PBAA/TPBAA) substantially between any double mutant and Col-0 ( **Figure 1B** ). These results show that the PBAA composition is tightly regulated in all mutants, similar to what we saw in our analysis of the single and triple mutants (Bagaza et al., 2024). We also measured all the free 20 proteogenic amino acid levels and calculated their composition ( **Amino Acid Dataset C, D** ) Unlike PBAA composition, the total levels of FAA (TFAA; i.e., the sum of all the FAA measured per mg) were affected across all double mutants, especially in *cruac* ( **Figure 1C** ). For the individual FAAs, Asn and histidine (His) levels increased the most with ~6.4- and 8.5-fold in *cruac* , respectively ( **Amino Acid Dataset C** ). FAA composition also changed significantly in the mutants, with Asn composition increasing the most at the expense of Glu ( **Amino Acid Dataset D** ). Still, the overall contribution of FAAs to the total amino acid pool remained very small. These results show that the FAAs are sensitive to perturbation and thus more plastic than PBAAs, however, their substantial alteration does not affect the levels or the composition of the PBAAs.

Next, we performed shotgun proteomics analysis on dry seeds of the double-knockout lines. The objective was to assess the response of the proteome to the eliminations of the different CRUs using mass spectrometry-based protein identification at the dry stage. We focused on the dry seed stage because it represents the final outcome of rebalancing on the seed proteome. The proteome analysis identified ~5000 proteins on average per genotype ( **Dry Proteome Dataset A** ). The significance of the proteome alteration for each mutant versus Col-0 was determined using a *t-test* followed by a False Discovery Rate (FDR) correction for multiple comparisons. We considered the mutant over Col-0 ratios statistically and biologically significant if the corrected *p* -value (FDR) was less than 5% and the fold change was above 1.2 or less than 0.8, which would represent a change of 20% or more. Our underlying hypothesis is that a 20% alteration is biologically relevant at the proteomics level. We identified a total of 94, 541, and 1166 proteins that were significantly differentially expressed (DEPs) in *cruab* , *crubc* , and *cruac* , respectively ( **Dry Proteome Datasets B, C, D** ). The highest proteomic alteration was observed in the *cruac* mutant, with a number of DEPs that is comparable to the triple mutant (Bagaza et al., 2024). The fact that the number of significant proteins in *cruac* (1166 DEPs) is comparable to the triple mutant (1828 DEPs) indicates that the combined loss of CRUA and CRUC has a profound effect on the seed proteome, similar to the complete absence of all the three CRUs. It is well known that CRUB is poorly transcribed and is the least abundant isoform compared to CRUA and CRUC (Withana-Gamage et al., 2013), which suggests that specific CRU proteins such as CRUA and CRUC may play a more critical role in maintaining proteome stability and that their combined effects are more essential for proteomic rebalancing than other combinations. For all the double mutants, however, most of the DEPs increased in their abundance, although such increase alone is not expected to account for the missing CRU proteins ( **Figure 1D ; Dry Proteome Dataset** ). Overall, these results suggest that the CRUs are not functionally redundant, and that each CRU is not replaceable by another in the *Arabidopsis* model system.

KEGG pathways showed that for the DEPs that increased in abundance, ribosome, fatty acid degradation, tryptophan metabolism, β-alanine metabolism, and photosynthesis-antenna proteins pathways were enriched in *cruac* , while proteasome was the only pathway that was enriched in *cruac* ( **Figure 1E** ). For the DEPs, that decreased in abundance, *cruac* had enriched terms that included biosynthesis of various plant secondary metabolites, nitrogen metabolism, and cyanoamino acid metabolism. These enriched pathways display a similar trend to those observed when evaluating the triple mutant in our most recent paper (Bagaza et al., 2024). KEGG pathways enrichment of the decreased proteins in *crubc* did not yield any significant terms. Likewise, *cruab* DEPs did not yield any significant enrichment ( **Figure 1E** ).

It was previously established that the CRU triple mutant was more sensitive to artificial aging and its seed protein content was more oxidized compared to Col-0 seeds (Nguyen et al., 2015). We recently showed that the CRU triple mutant has high levels of glutathione (GSH) and protein carbonylation (Bagaza et al., 2024). With this in mind, we measured the levels of GSH, a key antioxidant involved in maintaining the redox balance within cells, protecting them from oxidative stress, and regulating various cellular processes, in the dry seed of the various double mutants. GSH is the active, reduced form of glutathione that acts as a major antioxidant by neutralizing reactive oxygen species (ROS) and free radicals (Lushchak, 2012). GSH levels were significantly higher in *crubc* and *cruac* , while there was no significant difference for *cruab* ( **Figure 1F** ). GSSG is the oxidized form of glutathione that results when two GSH molecules are used to neutralize ROS, forming a disulfide bond. The levels of GSSG were only significant in *cruac* compared to Col-0 (one-way ANOVA) ( **Figure 1G** ). Significant elevation of GSSG levels in *cruac* suggests that this mutant is undergoing more substantial oxidative stress and consuming more GSH in the process, potentially explaining why this mutant showed the highest level of proteomic alterations. Then, we assessed the cellular redox state by using the commonly used ratio of GSH to GSSG. In general, more GSH reflects less stress, while more GSSG suggests oxidative stress (Zitka et al., 2012). However, comparison of the GSH/GSSG ratios for all double mutants revealed no significant change ( **Figure 1H** ). The observation that the *cruac* mutant exhibits high oxidative stress but manages to maintain GSH/GSSG balance further suggests that SSPs are integral to buffering the seed against oxidative damage. Even when certain SSPs are missing, the system can compensate to some extent, protecting the seed from deterioration. This indicates that the increase in ROS was mitigated successfully by all the double mutants. These results corroborate previous work that oxidation is involved in seed deterioration and that SSPs provide a buffering mechanism to combat oxidative stress (Nguyen et al., 2015; Bagaza et al., 2024).

To further confirm the role of SSPs in the redox buffering system, we measured the level of protein carbonylation across the double mutants. Protein carbonylation is one of the most widely used biomarkers of oxidative damage to proteins. The *cruac* mutant had a significantly higher level of carbonylation compared to all the double mutants ( **Figure 1I** ). Despite a stable GSH/GSSG ratio in *cruac* , the higher carbonylation suggests that while the glutathione system may be compensating for ROS to some extent, it is not fully preventing oxidative damage to proteins. The significantly higher carbonylation in *cruac* compared to other double mutants emphasizes the critical role of certain CRUs in protecting the seed proteome from oxidative damage. Since CRUB is the least abundant isoform (Withana-Gamage et al., 2013), this explains why double mutants that have eliminated CRUB, but retained the more abundant CRUA and CRUC proteins, did not show the same level of oxidative damage. This suggests that CRUA and CRUC are particularly important for maintaining redox homeostasis and preventing protein oxidation, which is linked to seed viability and stability.

To maintain proteomic integrity, the seed may need to synthesize new proteins to replace those that have been oxidized. This could lead to increased protein turnover and alterations in the overall proteomic composition that we observed in shotgun proteomics, which are hallmarks of proteomic rebalancing. Since each CRU protein has a unique and non-redundant function, their absence triggers distinct proteomic responses. Proteomic rebalancing occurs as the seed attempts to compensate for the loss of these specific proteins by adjusting the expression of other proteins. If CRU proteins were functionally redundant, the loss of one would be easily compensated by another, and rebalancing would be minimal. However, the distinct role of each protein indicates that the absence of any SSP will affect the seed proteome in a unique way, necessitating a tailored rebalancing response.

## Methods

**Plant Material and growth conditions**

The *Arabidopsis thaliana* mutant lines were generously provided by Dr. Dwayne D. Hegedus from Agriculture and Agri-Food Canada, Saskatoon, Saskatchewan S7N 0X2, Canada and consist of double mutants that are combinations of T-DNA insertion lines Salk_00266 ( *crua* ), Salk_045987) ( *crub* ), and GABI_283D09 *(cruc* ) targeting CRUA (At5g44120.3), CRUB (At1g03880.1), and CRUC (At4g28520.1) (Withana-Gamage et al., 2013). Seeds from these mutants and Col-0 were grown in the growth chamber at 24°C/22°C (day/night) under long-day conditions (16 h of light/8 h of dark). Watering was provided as needed and seeds were harvested at full maturation.

**Total protein analysis**

Protein extraction followed the Hurkman and Tanaka, 1986 method as described by Yobi et al., 2020. Total protein was quantified using the Pierce 600 kit (Thermo Scientific) according to the manufacturer’s instructions. Briefly, 5 mg of seed were extracted with Tris-HCl buffered phenol, and the protein pellet was resuspended in saline with sodium azide. Absorbance was measured at 600 nm using a plate reader, and protein content was determined using a BSA standard curve, adjusting for dilution and sample weight.

**Amino acid analysis**

PBAAs were extracted as described in (Yobi and Angelovici, 2018; Yobi et al., 2020). Briefly, ~3 mg of dry seeds from six biological replicates (n = 6) were hydrolyzed with 6N HCl for 24 h at 110 ^o^ C and the released amino acids were analyzed as free amino acids. For the FAAs, we extracted with water containing 13 internal standards as detailed in (Yobi et al., 2020; Bagaza et al., 2024).

**Glutathione analysis**

Glutathione was analyzed using the Glutathione Assay kit from Cayman Chemical (Ann Arbor, MI) following the manufacturer’s protocol as detailed in (Bagaza et al., 2024).

**Protein carbonyl content**

To determine protein carbonylation in the CRU mutants, we used the Protein Carbonyl Colorimetric Assay Kit from Cayman Chemicals (Ann Arbor, MI) following the manufacturer’s instructions as detailed in (Bagaza et al., 2024).

**Proteome analysis**

Protein extraction was performed as described in detail in (Yobi et al., 2020; Bagaza et al., 2024).

**Statistical and bioinformatics analysis**

For all GO enrichment analysis, we used ShinyGO v0.8 with Arabidopsis (TAIR 10) as a model and 0.05% FDR cutoff (Ge et al., 2020). The proteins detected in the dry seed proteomics study were used as a background for enrichment of the significant proteins. Enrichment bar plots were generated using ggplot2 v3.4.4 and RColorBrewer v1.1-3. For statistical analyses, unpaired t-test for amino acid analysis in the Dataset, one-way ANOVA with Dunnet’s post hoc test was performed using Excel and using R multcomp package v1.4.26 in R v4.3.2.

## Data Availability

Description: Amino acid analysis. Resource Type: Dataset. DOI: https://doi.org/10.22002/gqqxk-ysx83 Description: Dry seed proteome. Resource Type: Dataset. DOI: https://doi.org/10.22002/s7z8x-7j916
